# Naturally Occurring Flavonol, Quercetagetin 5,6,7,3′,4′-Pentamethyl Ether (Marionol), as a Nontoxic Plant-Based Fluorescent Probe for Rapid, Sensitive, and Selective Detection of Cu^2+^ in Water

**DOI:** 10.1021/acsomega.4c09069

**Published:** 2024-11-12

**Authors:** M. Deniz Yilmaz, Safaa Altves, Sundus Erbas-Cakmak

**Affiliations:** † Department of Basic Sciences, Faculty of Engineering, Necmettin Erbakan University, 42140 Konya, Turkey; ‡ BITAM-Science and Technology Research and Application Center, Necmettin Erbakan University, 42140 Konya, Turkey; § Department of Molecular Biology and Genetics, Faculty of Science, Necmettin Erbakan University, 42090 Konya, Turkey

## Abstract

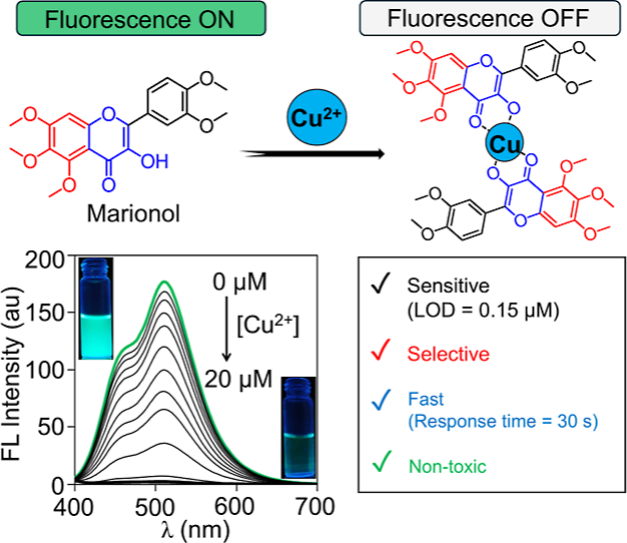

Herein, we report a naturally occurring flavonol, quercetagetin 5,6,7,3′,4′-pentamethyl ether (known as marionol), as a nontoxic fluorescent probe for rapid, sensitive, and selective detection of Cu^2+^ in water. The interaction between marionol and Cu^2+^ ions is studied by various state-of-the-art spectroscopic techniques such as ^1^H NMR, UV–vis, fluorescence, and high-resolution mass spectrometry (HRMS). Marionol shows strong fluorescence quenching in the presence of Cu^2+^, whereas other interfering cations such as Fe^3+^, Co^2+^, Al^3+^, Pb^2+^, Hg^2+^, Zn^2+^, Mg^2+^, Mn^2+^, Ca^2+^, Cu^+^, Ag^+^, Na^+^, and K^+^ produce negligible changes in fluorescence. The binding stoichiometry between marionol and Cu^2+^ is found to be 2:1, according to Job’s plot and HRMS analyses. The binding constant (*K*_a_) of Cu^2+^ with marionol is calculated to be 2.89 × 10^9^ M^–2^, and the limit of detection is found to be 0.15 μM. In addition, marionol is further used for the quantification of Cu^2+^ in natural spring water samples. The good recoveries (92–110% with RSD <5%) and its low toxicity to MCF-7 human breast cancer cells make marionol a unique plant-based fluorescent probe for the detection of Cu^2+^ ions.

## 1 Introduction

Copper is a crucial trace element in living organisms since it participates in many essential biological processes as an enzyme cofactor and an oxidation–reduction catalyst.^1,2^ Modern heavy industry and agriculture extensively use this unique metal; however, it is a strong environmental pollutant, so it should be taken seriously. In fact, the overdose of copper ions can cause serious neurodegenerative diseases, e.g., Alzheimer’s, Menkes, Prion, and Wilson’s.^3−5^ The permissible limit of 2 ppm (∼31 μM) for Cu^2+^ in drinking water was reported by the World Health Organization (WHO).^6^ Thus, detecting and monitoring Cu^2+^ ions in water samples is essential for human health and the environment.

Over the years, a variety of analytical techniques have been applied for the sensing of Cu^2+^ ions such as spectrophotometry, inductively coupled plasma mass spectrometry (ICP–MS), fluorescence spectrometry, atomic absorption spectroscopy, and electrochemical methods.^7−9^ Compared to other techniques, fluorescence spectrometry is a widely used analytical method for Cu^2+^ quantification due to its unique properties such as simplicity, cheapness, high sensitivity, and low limit of detection (LOD).^10−12^ In recent years, a great number of synthetic fluorescent probes have been developed for sensing Cu^2+^ ions;^7,10,13−23^ however, these artificially prepared probes have some limitations including long and multistep synthetic procedures, low solubility in water, poor bioavailability, and cell toxicity. Therefore, exploring new fluorescent molecules that address these issues is urgently needed.

Flavonols, a class of phytochemicals, are secondary metabolites of plants and are widely distributed in fruits, vegetables, and flowers.^24−26^ They possess unique excited-state intramolecular proton transfer (ESIPT)-based fluorescence properties,^27^ so they have been considered as nontoxic and biocompatible plant-based fluorophores for (bio)sensing and molecular imaging purposes.^28−35^ Few natural flavonol-based fluorescent probes for the detection of Cu^2+^ ions have already been reported in the literature so far. Natural quercetin was recently applied as a sensitive and selective fluorescent probe for the detection of Cu^2+^.^36−38^ The fluorescence of quercetin was quenched in the presence of Cu^2+^, and the probe showed a linear response to the concentration change of Cu^2+^. The LOD was found to be 0.1 μM. The binding stoichiometry was also determined by Job’s plot experiments, and the results confirmed that the stoichiometric ratio for the complexation of Cu^2+^ and quercetin is 1:1.^36^ Pant et al. utilized 3-hydroxyflavone for the fluorometric detection of Cu^2+^.^39^ The detection limit was reported as 1.54 μM, and a 1:1 binding stoichiometry was found between the probe and Cu^2+^ according to the Benesi–Hildebrand plots. Cao and co-workers reported that a natural isorhamnetin extracted from *Ginkgo biloba* leaves could be used for fluorometric detection of Cu^2+^.^40,41^ The interaction between isorhamnetin and Cu^2+^ caused a decrease in fluorescence proportional to the amount of Cu^2+^. The isorhamnetin–Cu^2+^ complex with a binding stoichiometry of 2:1 was very stable with an association constant of 1.79 × 10^6^ M^–1^. The detection limit was reported as 4 nM.^40^ Pitchumani et al. reported a phytochemical, biochanin A, as a fluorescent probe for Cu^2+^ with good selectivity and sensitivity.^42^ The LOD was calculated to be 1 μM, and the 1:1 complex formation was confirmed by ESI-MS and Job’s plot experiments. Although these naturally occurring flavonol-based detection systems for Cu^2+^ ions have been recently reported, their sensor performances need further improvement and the investigation of new biocompatible, nontoxic natural flavonols with unique fluorescent properties for detection of Cu^2+^ is of critical importance for human health. To our knowledge, research in this direction is limited.

**Scheme 1:**
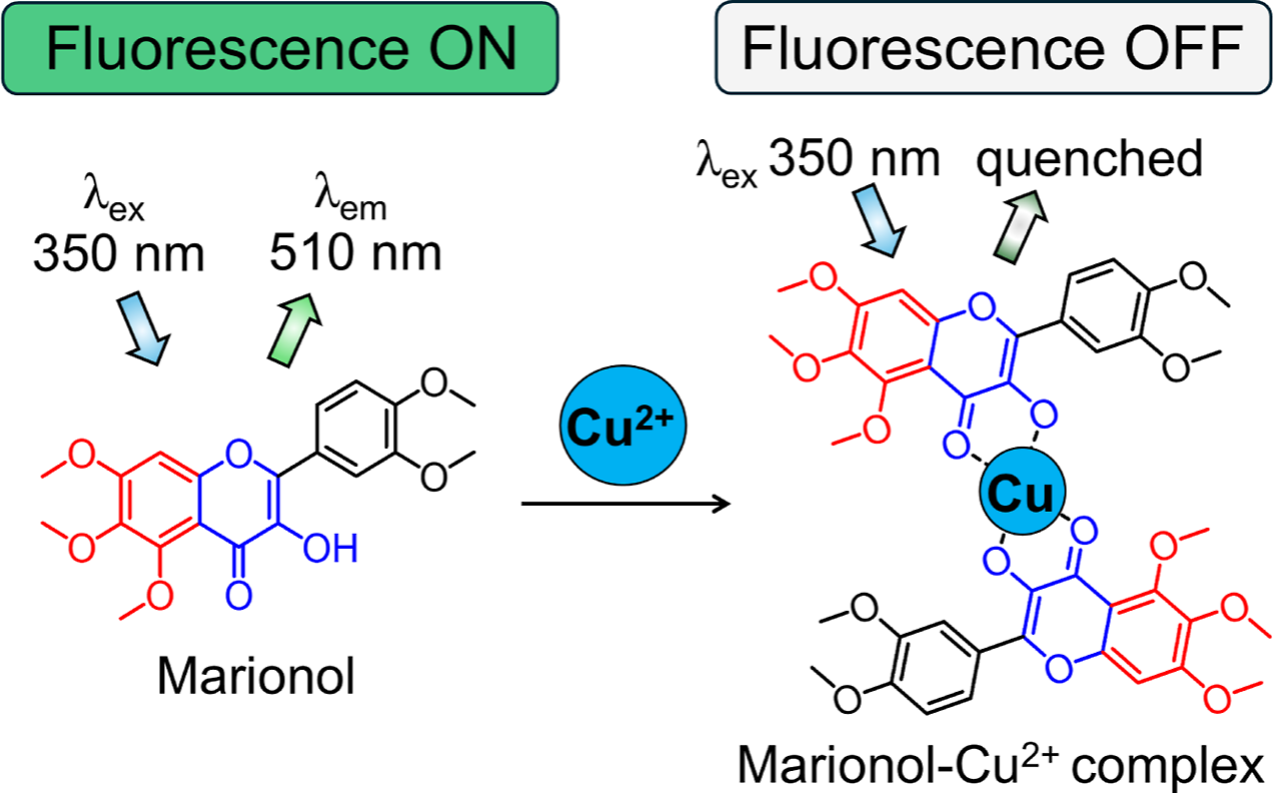
Chemical Structure of Marionol and the Proposed Sensing Mechanism

Herein, we report, for the first time, a naturally occurring unique flavonol, quercetagetin 5,6,7,3′,4′-pentamethyl ether, as a biocompatible and nontoxic fluorescent probe for rapid, sensitive, and selective detection of copper ions in water. To our knowledge, quercetagetin 5,6,7,3′,4′-pentamethyl ether, discovered first time in 1996 during the extraction of flavonoids of *Chromolaena odorata* leaves and it was assigned the name “marionol” in honor of Marion Dörr who isolated and characterized this plant flavonol,^43^ has not been used as a fluorescent probe to date. In our hands, fluorescent experiments revealed that marionol coordinated to Cu^2+^ in a 2:1 stoichiometric fashion and the fluorescence of marionol in water is quenched with Cu^2+^ (Scheme 1). It exhibited a rapid response (30 s) and a low detection limit (0.15 μM). The low cell toxicity of marionol makes it a promising plant-based probe for the detection of Cu^2+^ ions in real applications.

## 2 Experimental Section

### 2.1 Materials and Methods

All experiments were carried out at room temperature (25 °C) and normal atmospheric pressure (1 atm). All chemicals used in this study CuSO_4_·5H_2_O, CuI, AgNO_3_, NaCl, KCl, CaCl_2_, HgCl_2_, MgSO_4_, MnCl_2_·4H_2_O, PbCl_2_, Zn(OAc)_2_.2H_2_O, CoCl_2_·6H_2_O, FeCl_3_·6H_2_O, AlCl_3_, EDTA, PBS (phosphate buffered saline, pH 7.4), HCl, NaOH, DMSO-*d*_6_, and CDCl_3_ were purchased from commercial sources. Purified water (resistivity > 18 MΩ cm) was used in all experiments. Drinking water samples were purchased from a local market. Marionol was synthesized according to a previously described procedure (characterization data—^1^H NMR spectrum and high-performance liquid chromatography (HPLC) chromatogram—can be found in the Supporting Information, Figures S1 and S2).^44^

NMR spectra were recorded on a Bruker AvanceCore (400 MHz) spectrometer in the indicated solvent at room temperature. Chemical shifts are reported in parts per million (ppm) downfield from (CH_3_)_4_Si (TMS). High-resolution mass spectra were recorded with an Agilent 6530 Accurate-Mass Q-TOF LC/MS system. HPLC measurements were performed by a Shimadzu Prominence LC-20AT system. Briefly, 1.0 g of marionol was dissolved in 1.0 mL of acetonitrile. The solution was filtered through a 0.45 μm cellulose acetate membrane filter before being injected into the HPLC system. The experiment was carried out using a C18 column (Inertsil ODS-3, particle size 5 μm, column dimension 250 × 4.6 mm), operated at 25 °C. The solvent was a mixture of acetonitrile/water/*o*-phosphoric acid (50:50:0.1, v/v/v). The flow rate was set at 1 mL/min, and the injection volume was 10 μL. The UV detection was carried out at 350 nm. UV–vis absorption and steady-state fluorescence measurements were taken with Shimadzu UV3600i plus UV–vis–NIR and Agilent Cary Eclipse spectrophotometers, respectively. The excitation wavelength was 350 nm, and both excitation and emission slit widths were 5 nm for all measurements. The fluorescence quantum yields were calculated by a relative method with 4′,6-diamidino-2-phenylindole, DAPI, as the standard fluorescent dye (Φ_F_ = 0.045 in water). The fluorescence decay measurements were carried out with a Horiba Jobin-Yvon time-resolved fluorometer, Fluorolog FL-1057. A 390 nm Nano LED pulsed light source was used for the excitation. The instrument response was measured with an aqueous Ludox solution. The decays were analyzed with a multiexponential fitting function by iterative reconvolution and chi-square minimization.

### 2.2 Stock Solution of Marionol and Optical Measurements

The stock solution of marionol (1 mM) was prepared by dissolving 3.88 mg of marionol in 10 mL of acetonitrile and stored at +4 °C. Stock solutions of CuSO_4_·5H_2_O (1 mM) and interfering cations (1 mM each) were prepared in 10 mL of water at pH 7. The experiments were done by addition of the stock solutions of CuSO_4_·5H_2_O and interfering cations into a solution of 20 μM marionol (40 μL of a stock solution of marionol was added into 2000 μL of PBS containing 2% acetonitrile to obtain 20 μM marionol).

### 2.3 Cell Viability Assay and Cell Imaging

MCF-7 human breast cancer cells seeded on a 96-well plate were treated with marionol (0–320 μM) for 24 h in high-glucose Dulbecco’s modified Eagle medium supplemented with 10% fetal bovine serum (FBS) and 0.5% gentamycin with 5% CO_2_ in a humidified incubator at 37 °C. 3-(4,5-Dimethylthiazol-2-yl)-2,5-diphenyltetrazolium bromide (MTT, 0.25 mg/mL) was added, and cells were further incubated for 4 h in the dark. 100 μL of dimethyl sulfoxide was added to each well, the absorbance at 570 nm was recorded, and percent cell viabilities were estimated with respect to the untreated cell control group. All experiments were performed with at least three replicates, and the statistical analysis was done using GraphPad one-way ANOVA software. For fluorescence microscopy analysis, MCF-7 cells were seeded with a seeding density of 2 × 10^5^/well and incubated in the HG-DMEM medium supplemented with 10% FBS and 0.5% gentamycin for 24 h. Marionol (100 μM) was added to the cell medium, and the cells were further incubated for 2 h. Cells were fixed with a 4% formaldehyde solution, and fluorescence images were obtained using Zeiss LSM 900 confocal microscopy using a 385 nm LED light source and Axiocam 305 imaging device.

## 3 Results and Discussion

**Figure 1:**
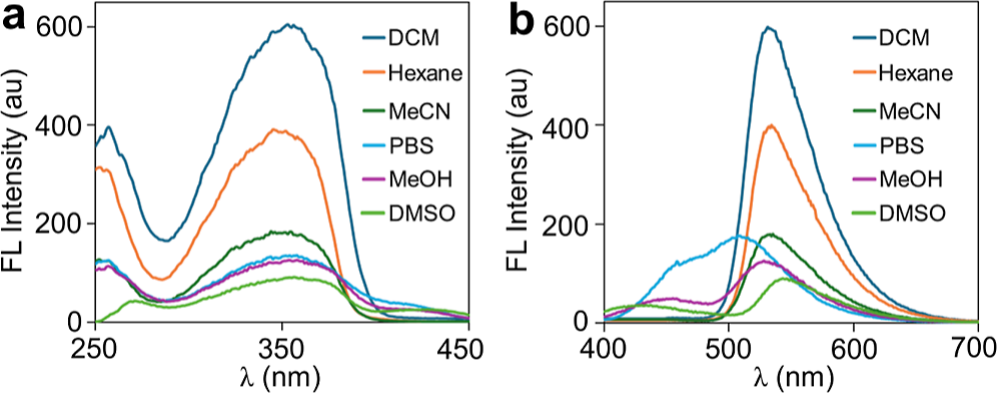
Excitation (a) and emission (b) spectra of marionol (20 μM) in different solvents (λ_exc_ = 350 nm).

Marionol is a yellow compound in its powder form and has an absorption band centered at 350 nm in PBS solution. The photophysical properties of marionol in different solvents including DMSO, MeOH, DCM, CH_3_CN, and hexane were examined by UV–vis and fluorescence measurements (Figure 1). Marionol has a strong absorption and excitation peak at around 350 nm in both organic solvents and PBS (Figure 1a). In addition, it displays green fluorescence in solvents used in this study at an excitation wavelength of 350 nm (Figure 1b). As can be seen in Table 1, both fluorescence intensity and quantum yield decrease as the solvent polarity increases due to the non-radiative transitions in polar solvents.^45^ In addition, a large Stokes shift is observed, which allows sensitive fluorescence detection by reducing self-quenching through self-absorption.

**Table 1:** Photophysical Characterization of Marionol

solvent	λ_max,abs_ (nm)	ε (M^–1^ cm^–1^)	λ_max,ex_ (nm)	λ_max,ems_ (nm)	Δλ_ST_ (nm)	Φ_F_^a^
dichloromethane	356	19,737	353	531	175	0.076
hexane	350	15,698	351	534	184	0.048
acetonitrile	352	19,358	350	535	183	0.02
phosphate-buffered saline (PBS)	357	18,642	356	510	153	0.014
methanol	357	18,797	358	528	171	0.014
dimethyl sulfoxide	355	18,623	356	542	187	0.012

a Reference compound: 4′,6-diamidino-2-phenylindole, DAPI (in water, Φ_F_ = 0.045).^46^

**Figure 2:**
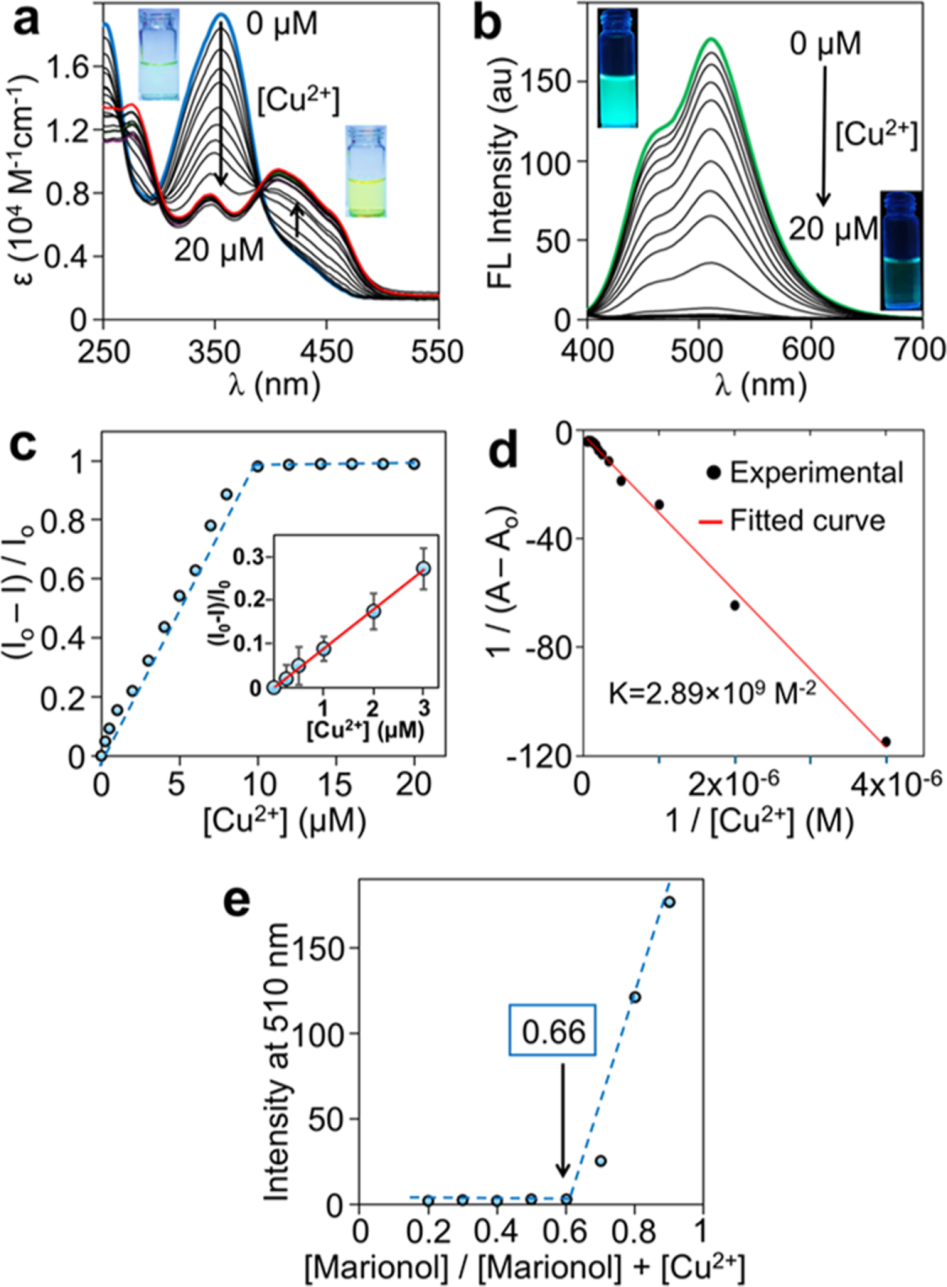
(a) Absorption titration spectra of marionol (20 μM in PBS buffer) in the presence of different concentrations of Cu^2+^. Inset: Photograph of the color change of marionol. (b) Fluorescence titration spectra of marionol (20 μM in PBS buffer) in the presence of different concentrations of Cu^2+^ (λ_ex_ = 350 nm). Inset: photograph of the emission change of marionol. (c) Change in the emission at 510 nm as a function of Cu^2+^ concentration. Inset: the plot of the emission at 510 nm against the concentration of Cu^2+^ ions (data points represent the mean of three independent measurements, *n* = 3). (d) Benesi–Hildebrand curve fitting data. (e) Job’s plot for determining the stoichiometry of the complexation between marionol and Cu^2+^.

To show the sensing ability of marionol as a plant-based molecular probe, UV–vis and fluorescence titration experiments were performed by adding Cu^2+^ ions (0–20 μM) to a PBS solution. The successive addition of Cu^2+^ ions to marionol (20 μM) resulted in a decrease in absorbance at 350 nm and an increase at 410 nm with an isosbestic point at 390 nm (Figure 2a). The color of the solution was changed from colorless to light yellow. Besides, the fluorescent intensity at 510 nm significantly decreased (quenched) when excited at 350 nm (Figure 2b). A plot of the fluorescence changes versus Cu^2+^ concentration increased linearly and reached a saturation point after the addition of 10 μM Cu^2+^ in which there was no further change in fluorescence, indicating a 2:1 coordination between marionol and Cu^2+^ (Figure 2c). Time-resolved fluorescence lifetime measurements were performed to validate the turn-off sensing mechanism. The average fluorescence lifetime of marionol in the absence of Cu^2+^ was measured to be 3.6 ns. On the other hand, it decreased to 2.5 ns in the presence of Cu^2+^ (Figure S3). By using the Benesi–Hildebrand method (eq 1 and Figure S4), the association constant of the marionol–Cu^2+^ complex was calculated and found to be 2.89 × 10^9^ M^–2^, indicating the strong coordination of marionol to Cu^2+^ (Figure 2d).^47^
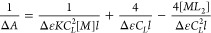
1

From the data of Figure 2c, the LOD was calculated to be 0.15 μM based on the signal-to-noise ratio (S/N = 3),^48^ which is nearly 200 times lower than the maximum permissible limit for Cu^2+^ in drinking water (∼31 μM) reported by the WHO.^6^ The binding stoichiometry was further studied by Job’s method.^49^ The emission intensity at 510 nm was plotted against the molar fraction of marionol, and the results confirmed a 2:1 binding between marionol and Cu^2+^ where two marionol coordinated to one Cu^2+^ ion (Figure 2e).

HRMS analysis was carried out to demonstrate the 2:1 binding event. The peak at *m*/*z* = 860.1122 (calcd = 860.1348) assigned to [(2 × marionol^–1^) + Cu^2+^ + Na^+^]^+^ proved 2:1 binding between marionol and Cu^2+^ (Figure S5). ^1^H proton NMR spectroscopy measurements were carried out to understand the binding mechanism. As shown in Figure S6, the proton signal of the –OH group at δ = 9.1 ppm broadened and almost disappeared upon the addition of Cu^2+^ ions and other peaks remained unchanged but broadened due to the paramagnetic nature of Cu^2+^ ions. These findings indicate that Cu^2+^ most probably binds to marionol through one hydroxyl- and one carbonyl-oxygen atom.

**Figure 3:**
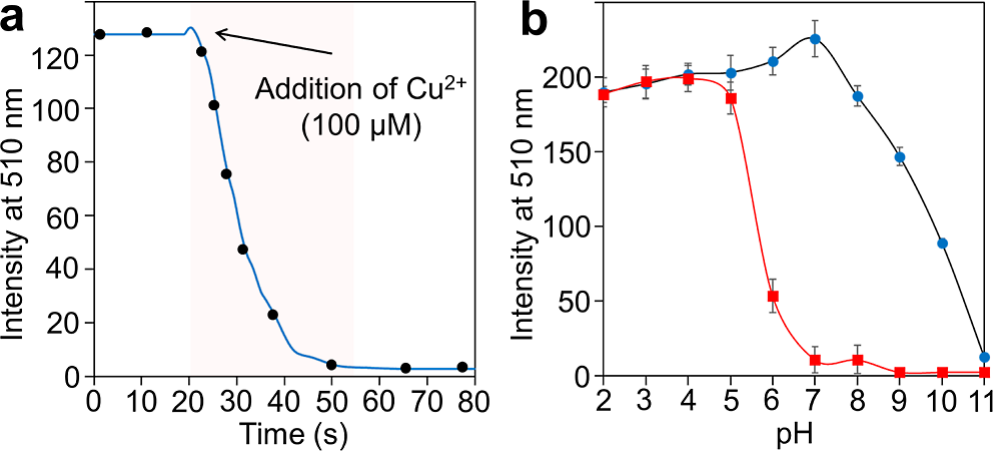
(a) Effect of time on the emission of marionol (20 μM) at 510 nm in the presence of 5 equiv of Cu^2+^ (100 μM) in PBS buffer (λ_ex_ = 350 nm). (b) Effect of pH on the emission of marionol (20 μM) without (●, blue) and with (■, red) Cu^2+^ (10 μM).

The response time of a probe to an analyte is a crucial performance characteristic. Hence, the time-dependent fluorescence response of marionol to Cu^2+^ was studied. The fluorescence of the solution was completely quenched within 30 s upon the addition of Cu^2+^ (Figure 3a). This fast response time makes marionol a promising natural probe for the fast fluorometric detection of Cu^2+^ ions in water. The effect of the pH on probe performance is another important parameter. Therefore, pH titration of marionol in the presence and absence of Cu^2+^ ions was performed. In Figure 3b, the fluorescence intensity of marionol at 510 nm is high at acidic and near neutral pH values; however, the intensity decreases at high pH values (>7) because of the deprotonation of the hydroxy group of marionol. In contact with Cu^2+^ ions, the intensity is high at acidic pH values in the range of pH 2–5 but quenches after this range. The results indicate that the neutral solutions (pH around 7) are the best working environments for marionol, and it is unique not only for sensing applications but also for cell studies.

**Figure 4:**
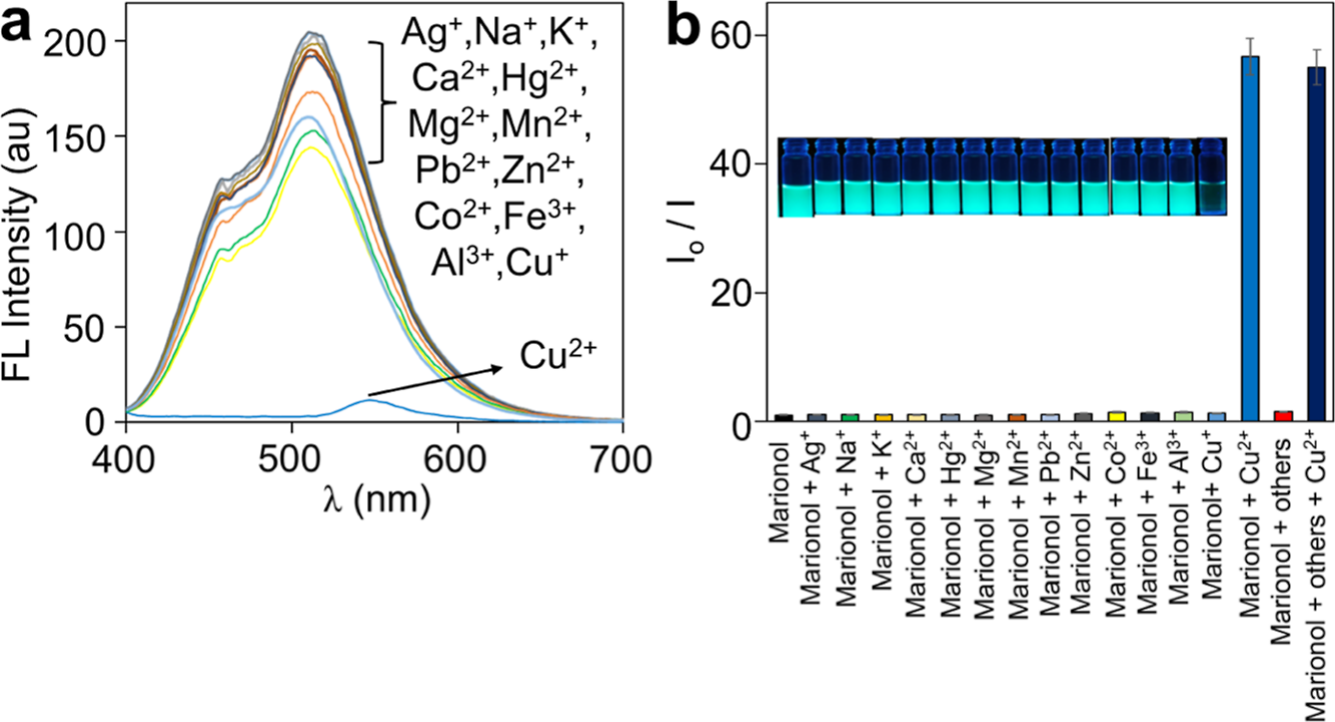
(a) Fluorescence spectra of marionol (20 μM) upon the addition of interfering cations (100 μM each) in PBS buffer solution (λ_ex_ = 350 nm). (b) Photographs of solutions under UV light and the corresponding bar plots in the absence and presence of interfering cations (data points represent the mean of three independent measurements, *n* = 3).

Marionol exhibited remarkable selectivity toward Cu^2+^ ions over other environmentally important cations such as Fe^3+^, Co^2+^, Al^3+^, Pb^2+^, Hg^2+^, Zn^2+^, Mg^2+^, Mn^2+^, Ca^2+^, Ag^+^, Cu^+^, Na^+^, and K^+^. As can be seen in Figures 4a and S7, negligible fluorescence quenching and absorbance changes in the presence of interfering cations (100 μM each) were observed. On the other hand, only the presence of Cu^2+^ (20 μM) led to a strong fluorescence decrease and absorbance shift under the same experimental conditions (Figures 4b and S7). These results indicate that the other environmental and biological cations do not markedly interfere with the coordination of marionol to Cu^2+^. We hypothesized that Cu^2+^ coordinates with carbonyl oxygen and neighboring hydroxyl oxygen which are involved in complexation with Cu^2^,^+^ leading to fluorescence quenching since Cu^2+^ is a powerful fluorescence emission quencher due to its paramagnetic nature (Scheme 1).^12^ The geometric configuration of hydroxy- and carbonyl-moieties at positions 3 and 4 creates a well-defined binding pocket for Cu^2+^, and this pocket coordinates with Cu^2+^ with ease rather than the other cations due to the different ionic radius.^50^

**Figure 5:**
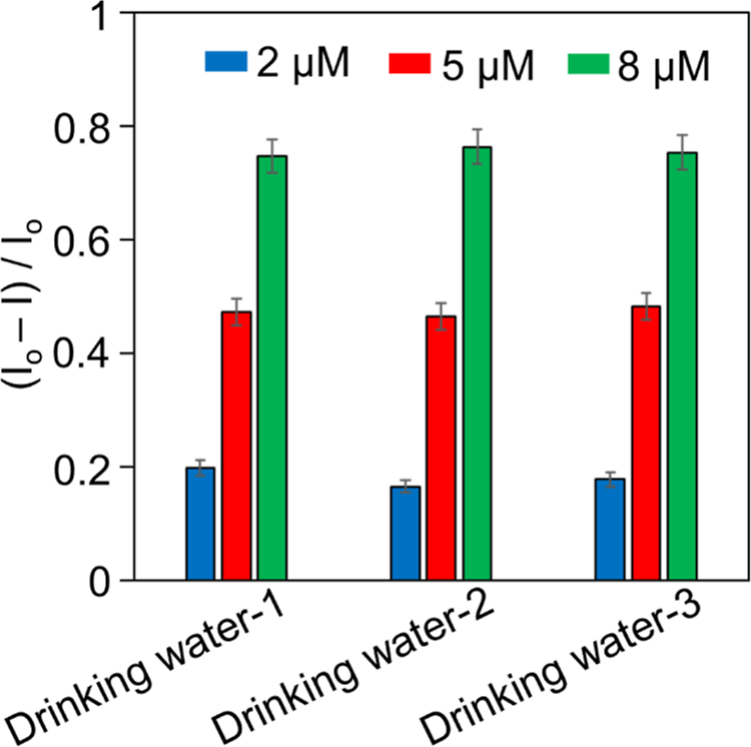
Fluorescence change at 510 nm of marionol (20 μM) in natural spring water samples. The samples were spiked with different amounts of Cu^2+^ (2, 5, 8 μM) (data points represent the mean of three independent measurements, *n* = 3).

The sensor performance of marionol as a practical fluorescent probe was tested in three commercially available natural spring water samples. The samples were treated with different amounts of Cu^2+^ (2, 5, and 8 μM), and the fluorescence intensity of solutions was monitored at 510 nm. By using the calibration curve obtained from Figure 2c, the final concentrations of Cu^2+^ in real water samples were calculated (Figure 5), and the results are summarized in Table 2. The average recoveries were obtained in the range of 92–110% with relative standard deviations of <5.0% for all water samples. The results clearly show that marionol can be directly applied to the detection and quantification of Cu^2+^ in real water samples.

**Table 2:** Determination of Cu^2+^ Ions in Natural Spring Water Samples

samples	added Cu^2+^ (μM)	measured Cu^2+-^(μM)	recovery (%)	RSD (%) (*n* = 3)
drinking water 1	0	not found		
	2	2.2	110	0.9
	5	5.3	105	1.5
	8	8.3	104	2.9
drinking water 2	0	not found		
	2	1.8	92	1.1
	5	5.2	103	4.4
	8	8.5	106	1.6
drinking water 3	0	not found		
	2	1.9	95	2.6
	5	5.4	107	1.0
	8	8.4	105	4.0

**Figure 6:**
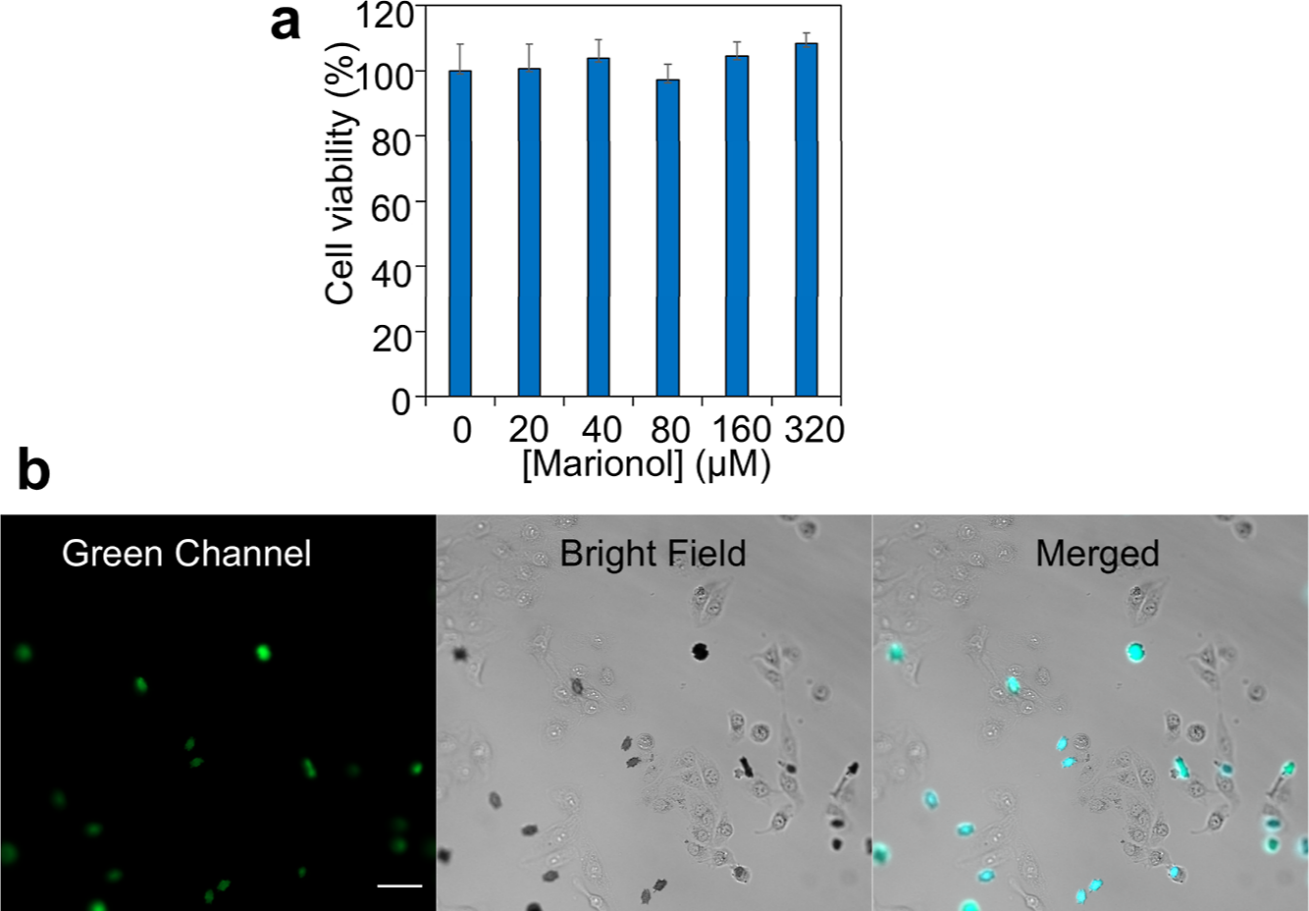
(a) Cell viability of MCF-7 human breast cancer cells incubated with various concentrations of marionol. (b) Confocal fluorescence microscopy images of MCF-7 human breast cancer cells incubated with marionol (100 μM). Scale bar: 50 μm.

To explore the cellular toxicity of marionol, an MTT assay against MCF-7 human breast cancer cells was performed. As depicted in Figure 6a, the cytotoxic effects of marionol were not statistically significant on MCF-7 human breast cancer cell lines in the concentration range from 20 to 320 μM. By using confocal fluorescent microscopy, the cellular uptake and internalization of marionol were evaluated (Figure 6b). The comparison of the fluorescent channel (green) and bright-field analysis showed that the location of marionol emission cannot be merged with cells but out of cells. This result indicated that marionol cannot be internalized within MCF-7 human breast cancer cells. These observations suggest that the broad range of concentrations of marionol did not exhibit any considerable toxicity on MCF-7 cells in vitro, and it could be safely used as a plant-based fluorescent probe for Cu^2+^ in routine applications.

The detection performance of marionol toward Cu^2+^ was compared with previously reported natural flavonol-based fluorescent probes. For this purpose, a comparison table was prepared, and the results are presented in Table 3. In comparison with prior fluorometric systems, marionol shows comparable sensing performance among the reported systems, which demonstrates the great potential of marionol for the detection and quantification of Cu^2+^ ions in water samples with almost no cytotoxicity issues.

**Table 3:** Performance Comparison of Natural Flavonol-Based Fluorescent Probes for the Detection of Cu^2+^

probe	linear range (μM)	LOD (μM)	response time (s)	real sample analysis	ref
quercetin	0.2–3	0.1	not mentioned	pure water sample	(36)
quercetin–cyclodextrin	0.05–8.3	0.023	not mentioned	river and lake water, food samples	(37)
quercetin–pluronic F127	0.05–4	0.01	not mentioned	river water	(38)
3-hydroxyflavone	0–8	1.54	not mentioned	not mentioned	(39)
isorhamnetin	0.01–1.9	0.004	not mentioned	river and lake water, food samples	(40)
isorhamnetin–cyclodextrin complex	0.05–6	0.017	not mentioned	river and lake water, food samples	(41)
biochanin A	2–100 μM	1	not mentioned	not mentioned	(42)
quercetagetin 5,6,7,3′,4′-pentamethyl ether (marionol)	0–10	0.15	30	natural spring water	this study

## 4 Conclusions

In summary, we demonstrated that marionol, a naturally occurring plant flavonol, could be employed as a nontoxic fluorescent probe for rapid, sensitive, and selective detection of copper ions in water. Marionol showed a strong binding ability to Cu^2+^ preferentially over other potential interfering cations, a fast response (∼30 s), and a low detection limit (0.15 μM). In addition, marionol displayed remarkable performance in real samples, such as commercially available natural spring water samples with high recoveries. Furthermore, the cytotoxicity studies confirmed the low toxicity of marionol to human cells. We therefore believe that marionol can be safely used for the detection and quantification of Cu^2+^ ions under real conditions, and this study will build new roads to discover natural and safer versions of fluorescent probes that can be used in environmental sensors and optical devices.

## Supporting Information Available

^1^H NMR spectrum and HPLC chromatogram of marionol; time-resolved fluorescence decay curves of marionol and the marionol–Cu^2+^ complex; determination of the binding constant; and ^1^H NMR and HRMS spectra of the marionol–Cu^2+^ complex (PDF)
