# Supplementary material for: Naturally Occurring Flavonol, Quercetagetin 5,6,7,3′,4′-Pentamethyl Ether (Marionol), as a Nontoxic Plant-Based Fluorescent Probe for Rapid, Sensitive, and Selective Detection of Cu2+ in Water
Source: ACS Omega. 2024 Nov 12;9(47):47342–9. doi: 10.1021/acsomega.4c09069 (PMC11603241; doi:10.1021/acsomega.4c09069)
Supplement: Supplementary file 1 — ao4c09069_si_001.pdf [file ao4c09069_si_001.pdf]

A naturally occuring flavonol, Quercetagetin 5,6,7,3',4'-pentamethyl ether (Marionol), as a non-toxic plant-based fluorescent probe for rapid, sensitive, and selective detection of Cu^{2+} in water

M. Deniz Yilmaz,[ab*] Safaa Altves,[b] Sundus Erbas-Cakmak[bc*]

[a] *Department of Basic Sciences, Faculty of Engineering, Necmettin Erbakan University, 42140 Konya, Türkiye.*

E-mail: deniz.yilmaz@erbakan.edu.tr; yilmazdnz@gmail.com

[b] *BITAM-Science and Technology Research and Application Center, Necmettin Erbakan University, 42140 Konya, Türkiye.*

[c] *Department of Molecular Biology and Genetics, Faculty of Science, Necmettin Erbakan University, 42090 Konya, Türkiye.*

E-mail: sundus.erbascakmak@erbakan.edu.tr

[*]Corresponding authors

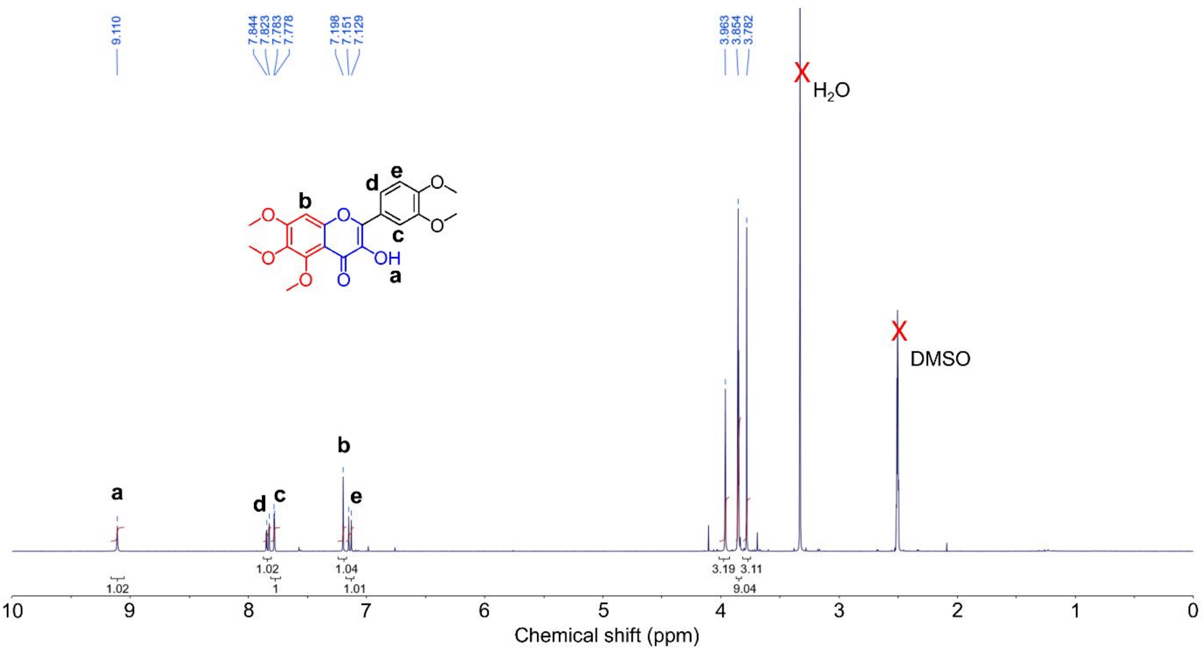

Figure S1. ^{1}H NMR spectrum of marionol (400 MHz, DMSO-d$_6$).

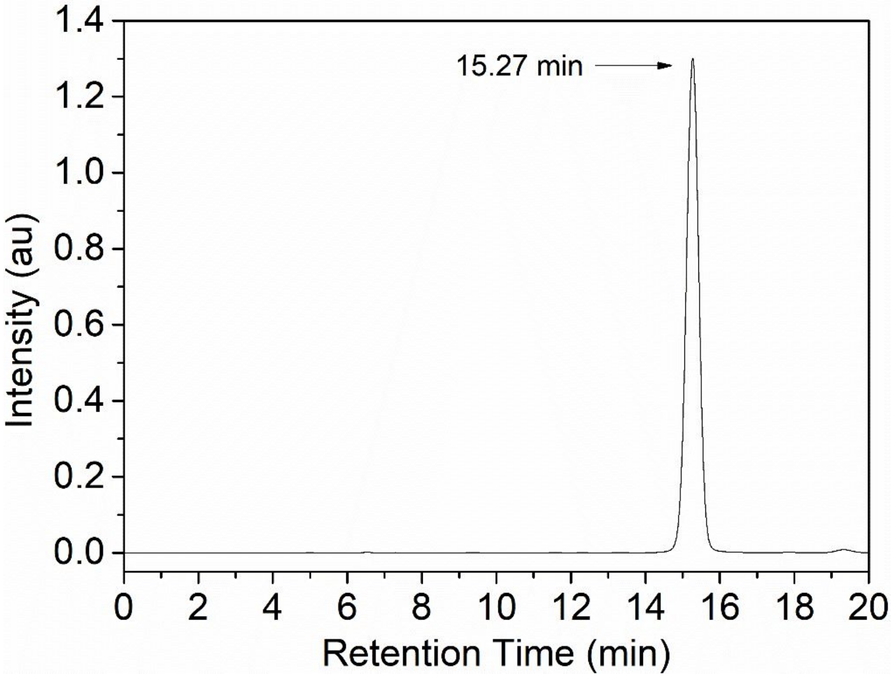

Figure S2. HPLC chromatogram of marionol (1 mg/mL in acetonitrile).

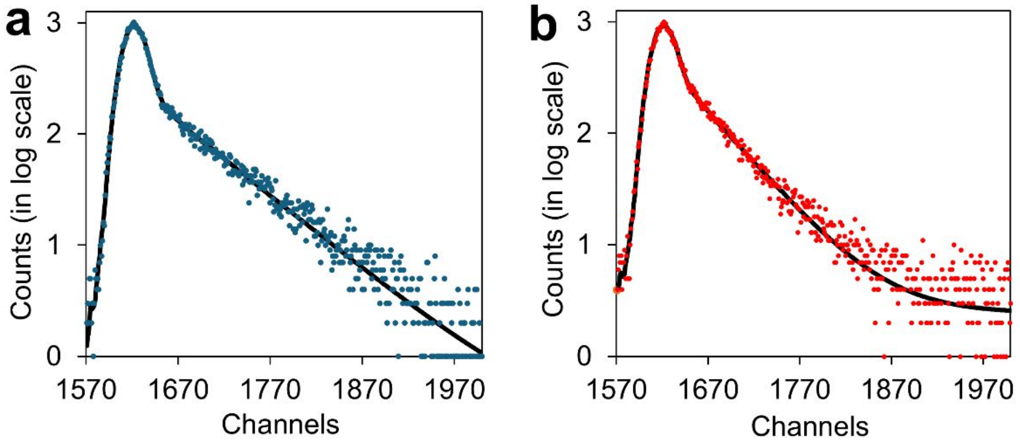

Figure S3. Time resolved fluorescence decay curves of marionol (100 μM in PBS) in the absence (a) and presence of Cu^{2+} (50 μM) (b).

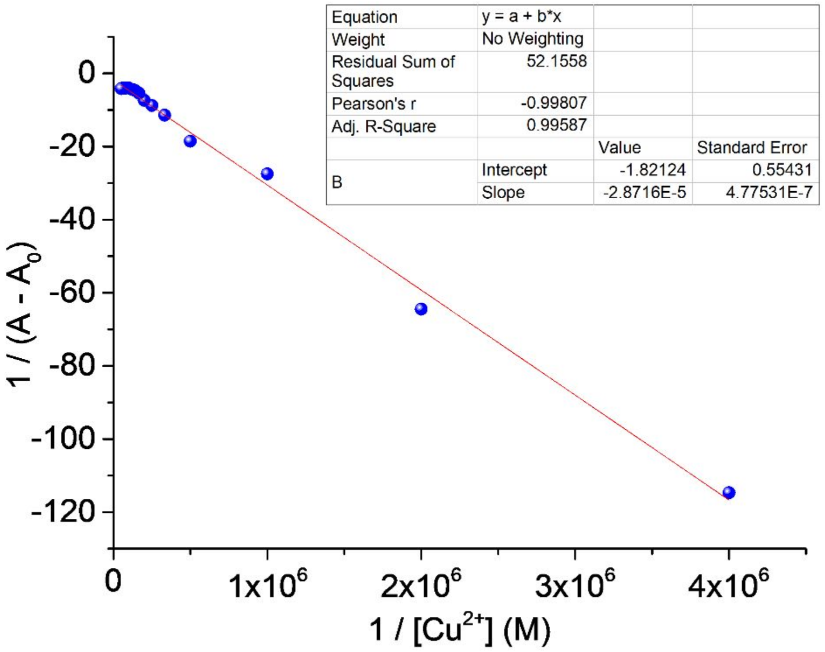

Figure S4. Benesi-Hildebrand linearization for binding constant calculation.

Benesi-Hildebrand Method

Determination of the binding constant

Using the stoichiometry 2:1 the association constant can be calculated as following:

$2L + M \leftrightarrows ML_2$ (1)

$K = [ML_2] / [L]^2[M]$ (2)

$C_0 = [L] + 2[ML_2] \rightarrow [L] = C_0 - 2[ML_2]$ (3)

$A = A_L + A_{ML2} = \varepsilon_L [L] l + \varepsilon_{ML2} [ML_2] l$ (4)

$C_0 = C_L$ (5)

Replacing eqn (3) and eqn (5) into eqn (4):

$A = \varepsilon_L C_L l - 2\varepsilon_L [ML_2] l + \varepsilon_{ML2} b [ML_2] l$ (6)

$A_{[M]=O} = \varepsilon_L C_L l$ and $\Delta\varepsilon = \varepsilon_{ML2} - 2\varepsilon_L$ (7)

$\Delta A = A - A_O = \varepsilon_{ML2} [ML_2] l - 2\varepsilon_L [ML_2] l = \Delta\varepsilon [ML_2] l$ (8)

Using the constant:

$\Delta A = \Delta\varepsilon K[L]^2[M]l = \Delta\varepsilon K [C_L - 2[ML_2]]^2[M]l$ (9)

$\Delta A = \Delta\varepsilon K[M] (C_L^2 - 4C_L [ML_2] + 4[ML_2]^2) l$ (10)

$\Delta A / l = \Delta\varepsilon K[M]C_L^2 - \Delta\varepsilon K[M]4C_L [ML_2] + \Delta\varepsilon K[M]4[ML_2]^2$ (11)

$\Delta A / l = \Delta\varepsilon K C_L^2[M] - \Delta A K 4C_L[M] + \Delta A K 4[ML_2] [M]$ (12)

$\Delta A / l = \Delta\varepsilon C_L^2 K[M] / 1+4K[M] (C_L - [ML_2])$ (13)

$1 / \Delta A = 1 / \Delta\varepsilon K C_L^2[M]l + 4 / \Delta\varepsilon C_L l - 4[ML2] / \Delta\varepsilon C_L^2 l$ (14)

Slope = $1 / \Delta\varepsilon K C_L^2 l$

The intercept = $4 / \Delta\varepsilon C_L l - 4[ML_2] / \Delta\varepsilon C_L^2$

$\varepsilon_L = 19083\ M^{-1}\ cm^{-1}$ at 350 nm *and* $\varepsilon_{ML2} = 8083.5\ M^{-1}\ cm^{-1}$ at 410 nm

$\Delta\varepsilon = \varepsilon_{ML2} - 2\varepsilon_L = -30082.5\ M^{-1}\ cm^{-1}$

$1 / \Delta A = 1 / \Delta\varepsilon K C_L^2 [M]l + 4 / \Delta\varepsilon C_L l - 4[ML_2] / \Delta\varepsilon C_L^2 l$

Slope = $1 / \Delta\varepsilon K C_L^2 l = -2.8716 x 10^{-5}$ M

$K = 1 / (-2.8716 x 10^{-5} M) (-30082.5\ M^{-1}\ cm^{-1}) (20 x 10^{-6} M)^2 (1\ cm)$

$K = 2.89 \times 10^9\ M^{-2}$

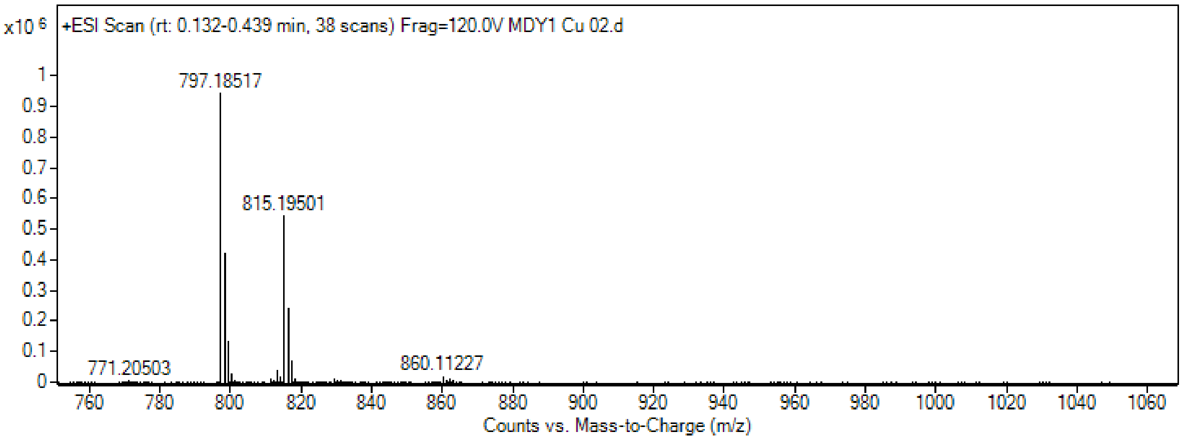

Figure S5. High-resolution mass (HRMS) spectra of marionol-Cu^{2+} complex.

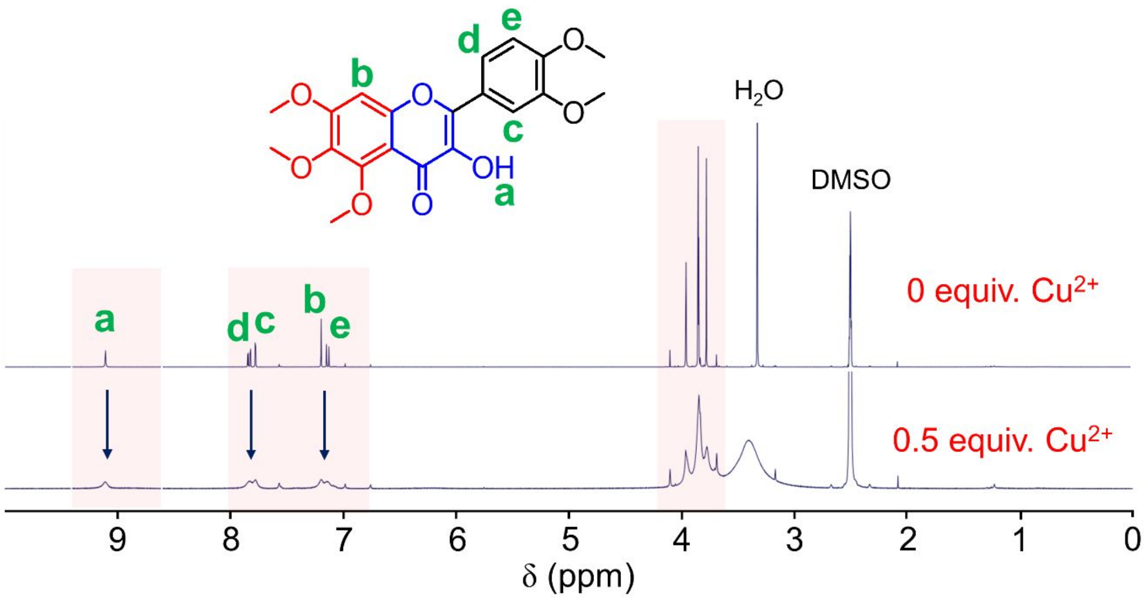

Figure S6. ^{1}H NMR spectra of marionol in the presence of 0.5 equivalent Cu^{2+} in DMSO-d_6.

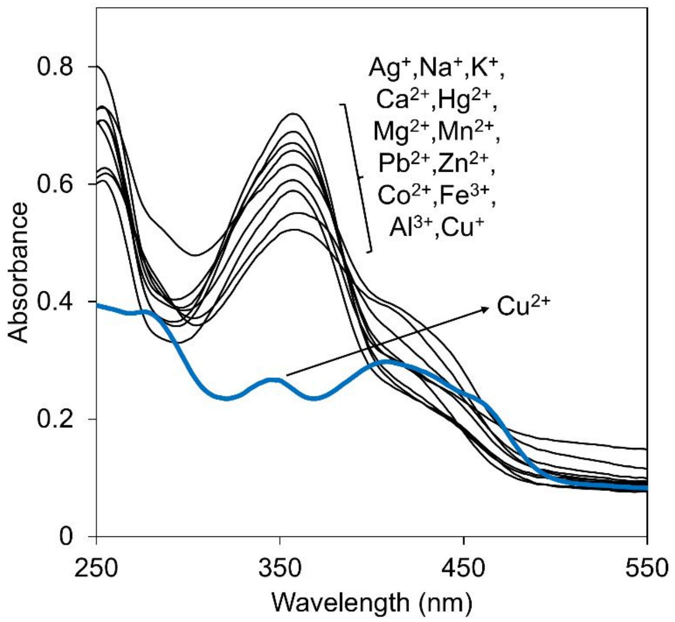

Figure S7. UV-vis spectra of marionol (20 μM) upon the addition of interfering cations (100 μM each) in PBS buffer solution.
